# Preventing the Adverse Effects of SARS-CoV-2 Infection and COVID-19 through Diet, Supplements, and Lifestyle

**DOI:** 10.3390/nu14010115

**Published:** 2021-12-28

**Authors:** Ronan Lordan, William B. Grant

**Affiliations:** 1 Institute for Translational Medicine and Therapeutics (ITMAT), Perelman School of Medicine, University of Pennsylvania, Philadelphia, PA 19104-5158, USA; 2 Sunlight, Nutrition, and Health Research Center, P.O. Box 641603, San Francisco, CA 94164-1603, USA; williamgrant08@comcast.net

Severe acute respiratory syndrome coronavirus 2 (SARS-CoV-2), the pathogen responsible for the coronavirus disease 2019 (COVID-19) and the ongoing worldwide pandemic, has cost the lives of almost 5.4 million people and infected over 276 million worldwide as of December 2021 [1]. While great strides have been made to produce and repurpose therapeutics, develop novel vaccines, and promote non-pharmacological interventions to reduce disease burden, parts of the world are now entering their fifth pandemic wave. An underappreciated mitigation strategy has been the role of preventing the adverse effects of COVID-19 by promoting healthy lifestyle patterns in conjunction with non-pharmacological interventions. This is even more important in parts of the world that are disadvantaged by their lack of access to vaccines. Moreover, additional protections via dietary and lifestyle changes may improve overall health. Indeed, it is well established that poor host nutritional status is a potential risk factor for severe respiratory diseases and comorbidities such as diabetes, hypertension, and obesity, which all increase the risk for severe disease, hospitalization, and death in COVID-19 patients [2,3].

In this Special Issue, we provided a platform for scientists to submit their research investigating nutritional status, the potential of dietary and lifestyle alterations, and the use of supplements in relation to COVID-19 outcomes. In this editorial, we present the advances this Special Issue has brought to fruition in the battle against the coronavirus pandemic.

At the beginning of the pandemic, multiple lines of evidence suggested a potential link between vitamin D and SARS-CoV-2 infection and COVID-19. AlSafar and colleagues [4] examined 25(OH)D levels in serum samples taken upon admission in 464 hospitalized COVID-19 patients in relation to patient outcomes in the United Arab Emirates (UAE). They determined that 25(OH)D < 12 ng/mL was significantly associated with 2.58-fold (95% CI, 1.01, 6.62) increased risk of COVID-19 mortality following adjustment for age, comorbidities or sex (*p* = 0.048). Indeed, Karanova et al. [5] presents further data in accordance with AlSafar et al. [4], which supports the notion that 25(OH)D deficiency is common among hospitalized COVID-19 patients. In their study, 90 out of 133 Russian COVID-19 patients were either 25(OH)D deficient or insufficient. Karanova et al. [5] also determined that 25(OH)D levels between approximately 11–12 ng/mL was the threshold value for increased risk of severe COVID-19 disease and mortality. Both studies add to a growing literature demonstrating that having sufficient 25(OH)D levels may be of critical importance and a predictor of coronavirus patient outcomes.

Considering these findings, one of the most important issues regarding COVID-19 is how to treat patients. In the article from Saudi Arabia, a small-scale randomized controlled trial was conducted to assess the effects of vitamin D supplementation on COVID-19 patients shortly after symptoms arose [6]. The trial involved 69 SARS-CoV-2-positive patients hospitalized with mild to moderate COVID-19 between 29 July and early September 2020. They were randomized to receive either 1000 or 5000 IU of vitamin D3 daily for 14 days. Most of the descriptive characteristics and symptoms were not significantly different between the two arms. Mean baseline 25(OH)D concentration was 63 ± 3 nmol/L in the 1000 IU arm and 53 ± 3 nmol/L in the 5000 IU arm, while mean 25(OH)D concentrations of 60 nmol/L were achieved in the 1000 IU arm and 63 nmol/L in the 5000 IU arm. However, those in the 5000 IU arm were younger (46 ± 15 vs. 54 ± 12 years, *p* = 0.03) and had lower BMI (28 ± 7 vs. 32 ± 7 kg/m^2^, *p* = 0.02). Two COVID-19 symptoms were resolved faster with 5000 IU/d: cough (6 ± 1 vs. 9 ± 1 days, *p* = 0.007) and ageusia (loss of taste) (11 ± 1 vs. 17 ± 2 days, *p* = 0.04). There were no significant differences in pre- and post-clinical parameters between the two arms other than D-dimer concentrations, which decreased in the 1000 IU arm but increased in the 5000 IU arm. One reason for limited beneficial effects of vitamin D supplementation in this trial may be that the low doses did not raise 25(OH)D concentrations rapidly enough to significantly affect the course of the disease. It is thought that providing much higher doses [7] or using calcifediol [25(OH)D] may have achieved more favorable clinical benefits [8].

Golabi and colleagues investigated the association between vitamin D and zinc status and progression of clinical symptoms among 53 outpatients in Iran infected with SARS-CoV-2 as well as 53 potentially non-infected participants [9]. Infected patients had non-significantly lower 25(OH)D concentrations than non-infected ones (26 ± 17 vs. 29 ± 16 ng/mL). There was a trend for lower 25(OH)D among patients with moderate illness than with asymptomatic or mild illness (19 ± 12 vs. 29 ± 18 ng/mL, *p* = 0.054). In terms of progression, patients with 25(OH)D > 20 ng/mL had a reduced progression compared to those with <12 ng/mL (OR = 0.19, *p* <0.001) as did those with 25(OH)D between 12 and 20 ng/mL compared to <12 ng/mL (OR = 0.3, *p* = 0.007). Infected patients had significantly lower serum zinc concentrations than non-infected patients (101 ± 18 vs. 114 ± 13 µg/dL). However, the difference between zinc concentrations for those with mild or no sign vs. moderate severity was not significant (*p* = 0.41). A study involving four SARS-CoV-2 or COVID-19 patients treated with high-dose zinc salts (23 to 150 mg/d) found significant improvements starting one day after treatment [10]. On the other hand, a larger study involving 58 patients given 50 mg/day zinc, 58 patients given 50 mg/d zinc plus 8 g/d ascorbic acid, and 48 patients given 8 g/d ascorbic acid, did not find any significant difference in secondary outcomes compared to 50 patients given the standard of care [6].

Many during the pandemic turned to seeking additional protections against SARS-CoV-2 and COVID-19 via the use of dietary supplement and nutraceuticals [11]. In this Special Issue, Puscion-Jakubik et al. examined the consumption of food supplements during the first three COVID-19 waves in Poland (spring and autumn 2020 and winter 2021) [12]. Approximately 300 participants responded to each questionnaire, with 80% of the respondents being women, the mean age was approximately 29 ± 10 years, and approximately 50% having medical or related education, and nearly all either working in an office or studying as a student. Thus, the survey does not relate to the general public. The authors reported that vitamin D was the most popular supplement during the second wave that started in September 2020, taken by 23% of the respondents during the first wave, 38% in the second wave, and 33% in the third wave. The mixture of office workers vs. students changed from 50% vs. 34% in the first wave to 39% vs. 51% in the second wave and 36% vs. 54% in the third wave, which seems to have affected the vitamin D supplementation findings.

Scientists have also had a keen interest to pursue the development of novel supplements or nutraceuticals to quell the spread of the pandemic and provide effective treatments for patients. In this Special Issue, two natural products, *Glycyrrhiza glabra* extract and hesperidin, have been assessed with a focus on inhibiting viral entry via angiotensin-converting enzyme 2 (ACE2) and transmembrane serine protease 2 (TMPRSS2), the key cellular proteins required for SARS-CoV-2 entry into mammalian cells.

Jezova et al. [13] aimed to harness the antiviral potential of glycyrrhizin, a saponin type molecule responsible for the sweet taste in *Glycyrrhiza glabra* (licorice) root. The authors show that a *Glycyrrhiza glabra* extract reduces ACE2 expression via inhibiting the activity of 11-β-hydroxysteroid dehydrogenase type-2 (11-β-HSD2) leading to the activation of the mineralocorticoid receptor (MR). The authors showed that supplementation of *Glycyrrhiza glabra* extract in a stress model in Sprague Dawley rats reduced the expression of ACE2 in target tissues where ACE2 is co-expressed with 11-β-HSD2 and MR, such as the ileum, versus tissues where co-expression of these proteins does not occur, such as the brain cortex. Although the authors were not able to conduct in vivo challenge studies with SARS-CoV-2 to assess if reduced expression of ACE2 in these target tissues reduces viral RNA copy number or disease severity, this study does show promise for further development and research.

Cheng et al. [14] investigated the binding capacity of hesperidin, a flavanone glycoside that naturally occurs in citrus fruits, and hesperitin, an aglycone metabolite of hesperidin, to ACE2 and TMPRSS2. Notably, the authors were able to show that both molecules could suppress the infection of VeroE6 cells by lentiviral pseudo-particles of wild type SARS-CoV-2 and variants with the D614G and 501Y.v2 (beta variant) mutations. Indeed, there was also a suppression of ACE2 and TMPRSS2 expression. In contrast, neither molecule affected SARS-CoV-2 viral proteases papain-like protease (PLpro) and 3-chymotrypsin-like protease (Mpro) despite molecular docking predictions. Despite these promising findings, it is important to interpret with caution as these experiments were conducted in silico and in vitro, which is not always translatable to efficacy in humans. Indeed, while veroE6 cells are commonly used for SARS-CoV-2 studies, they are not representative of the respiratory tract, the primary site of SARS-CoV-2 infection, and so findings should be interpreted with caution. Despite these caveats, it has been shown that 1 g daily of hesperidin given to symptomatic non-vaccinated COVID-19 patients in a randomized, double-blind, placebo-controlled trial appeared to modestly reduce some symptoms of COVID-19, including fever, cough, shortness of breath and anosmia, but much further study is required [15].

Other authors that contributed to this Special Issue took a much broader view of the potential nutritional requirements and supply chain issues that occurred during the pandemic. Currently more than 4.2 million children and 138,000 adults receive nutritious meals and snacks in the U.S. through the Child and Adult Care Food Program (CACFP) [16]. In the latest month for which data are available, August 2021, the total program cost was nearly $250 million [17]. In a review by Stephens and colleagues in this Special Issue [18], comparisons were made on how the COVID-19 pandemic affected operations for CACFP and non-CACFP in Arizona and Pennsylvania. Not surprisingly, CACFP sites were much more likely to offer “grab and go” meals, meal delivery, and distributed food boxes.

Indeed, maintaining a functional, hygienic, and sustainable food supply and distribution network during the pandemic has been vastly underappreciated. Filip et al. provide an in-depth review of the literature concerning the clinical outcomes of COVID-19 patients and how nutritional status and changes to the food chain, food hygiene, food security, and people’s dietary patterns during the coronavirus pandemic are interrelated and affect health [19]. Although fomites are no longer thought to significantly contribute to SARS-CoV-2 transmission, the authors also provide cautionary guidelines to the food industry for the processing, packaging, and distribution of food with the intention to limit the spread of SARS-CoV-2.

Another topic that was highlighted in this Special Issues was that responses to the COVID-19 pandemic can have unintended consequences. An example of this is found in the study of weight gain among school teachers in Long Island, NY, who switched from in person teaching to online teaching [20]. Teachers for grades kindergarten to 5 gained a mean weight of 4 ± 8 pounds, those who taught in middle school did not change mean weight (0 ± 11 pounds), while those who taught in high school lost weight (−1 ± 9 pounds). Some of the weight gain appears to be associated with emotional eating due to nothing to do, being bored, depressed, or discouraged, irritated, feeling anxious, feeling lonely, etc. Junk food including chips and ice cream had strong associations with weight gain. Exercise was associated with weight loss. This study should lead to additional studies that examine changes in weight due to changing environmental conditions and how to modify the effects. Another article examined emotional eating during the COVID-19 pandemic in Norway, finding that 54% of the respondents to an electronic survey reported emotional eating, with higher rates among women [21]. That led to higher intake of high-sugar foods and beverages. A paper published in 1996 reported that Type A women in Northern Ireland had a weak positive association with sugar and alcohol intake, as opposed to men, who had a significant association with fat and protein intake as well as beef, cheese, yoghurt, and chips [22]. An earlier article in Nutrients studied the effects of weight gain during pregnancy associated with emotional eating [23]. The authors suggested the need for psychosocial and nutritional education and interventions during pregnancy checkups.

As 2022 draws closer, the COVID-19 pandemic is not over and will likely affect everyday life for the foreseeable future. Despite the successful development and distribution of vaccines in the Western world, many regions have yet to receive adequate supply of vaccines. Therefore, the implementation of efficacious non-pharmacological interventions coupled with the promotion of healthy dietary and lifestyle patterns may promote overall health and reduce one’s risk of infection, disease, and death as a result of the SARS-CoV-2 pandemic. Evidentially, further extensive, and broad-ranging research is required to understand how the majority of the adverse effects of SARS-CoV-2 infection and COVID-19 could be prevented through diet, supplements, and lifestyle.

## References

[1] John Hopkins University John Hopkins University & Medicine: Coronavirus Resource Center. https://coronavirus.jhu.edu/map.html.

[2] Zabetakis I., Lordan R., Norton C., Tsoupras A. (2020). COVID-19: The inflammation link and the role of nutrition in potential mitigation. Nutrients.

[3] Im J.H., Je Y.S., Baek J., Chung M.-H., Kwon H.Y., Lee J.-S. (2020). Nutritional status of patients with COVID-19. Int. J. Infect. Dis..

[4] AlSafar H., Grant W.B., Hijazi R., Uddin M., Alkaabi N., Tay G., Mahboub B., Al Anouti F. (2021). COVID-19 Disease Severity and Death in Relation to Vitamin D Status among SARS-CoV-2-Positive UAE Residents. Nutrients.

[5] Karonova T.L., Andreeva A.T., Golovatuk K.A., Bykova E.S., Simanenkova A.V., Vashukova M.A., Grant W.B., Shlyakhto E.V. (2021). Low 25(OH)D Level Is Associated with Severe Course and Poor Prognosis in COVID-19. Nutrients.

[6] Sabico S., Enani M.A., Sheshah E., Aljohani N.J., Aldisi D.A., Alotaibi N.H., Alshingetti N., Alomar S.Y., Alnaami A.M., Amer O.E. (2021). Effects of a 2-Week 5000 IU versus 1000 IU Vitamin D3 Supplementation on Recovery of Symptoms in Patients with Mild to Moderate COVID-19: A Randomized Clinical Trial. Nutrients.

[7] Gönen M.S., Alaylıoğlu M., Durcan E., Özdemir Y., Şahin S., Konukoğlu D., Nohut O.K., Ürkmez S., Küçükece B., Balkan İ.İ. (2021). Rapid and Effective Vitamin D Supplementation May Present Better Clinical Outcomes in COVID-19 (SARS-CoV-2) Patients by Altering Serum INOS1, IL1B, IFNg, Cathelicidin-LL37, and ICAM1. Nutrients.

[8] Entrenas Castillo M., Entrenas Costa L.M., Vaquero Barrios J.M., Alcalá Díaz J.F., López Miranda J., Bouillon R., Quesada Gomez J.M. (2020). Effect of calcifediol treatment and best available therapy versus best available therapy on intensive care unit admission and mortality among patients hospitalized for COVID-19: A pilot randomized clinical study. J. Steroid Biochem. Mol. Biol..

[9] Golabi S., Adelipour M., Mobarak S., Piri M., Seyedtabib M., Bagheri R., Suzuki K., Ashtary-Larky D., Maghsoudi F., Naghashpour M. (2021). The Association between Vitamin D and Zinc Status and the Progression of Clinical Symptoms among Outpatients Infected with SARS-CoV-2 and Potentially Non-Infected Participants: A Cross-Sectional Study. Nutrients.

[10] Finzi E. (2020). Treatment of SARS-CoV-2 with high dose oral zinc salts: A report on four patients. Int. J. Infect. Dis..

[11] Lordan R. (2021). Dietary supplements and nutraceuticals market growth during the coronavirus pandemic—Implications for consumers and regulatory oversight. Pharma Nutr..

[12] Puścion-Jakubik A., Bielecka J., Grabia M., Mielech A., Markiewicz-Żukowska R., Mielcarek K., Moskwa J., Naliwajko S.K., Soroczyńska J., Gromkowska-Kępka K.J. (2021). Consumption of Food Supplements during the Three COVID-19 Waves in Poland—Focus on Zinc and Vitamin D. Nutrients.

[13] Jezova D., Karailiev P., Karailievova L., Puhova A., Murck H. (2021). Food Enrichment with Glycyrrhiza glabra Extract Suppresses ACE2 mRNA and Protein Expression in Rats—Possible Implications for COVID-19. Nutrients.

[14] Cheng F.-J., Huynh T.-K., Yang C.-S., Hu D.-W., Shen Y.-C., Tu C.-Y., Wu Y.-C., Tang C.-H., Huang W.-C., Chen Y. (2021). Hesperidin Is a Potential Inhibitor against SARS-CoV-2 Infection. Nutrients.

[15] Dupuis J., Laurin P., Tardif J.-C., Hausermann L., Rosa C., Guertin M.-C., Thibaudeau K., Gagnon L., Cesari F., Robitaille M. (2021). Fourteen-days Evolution of COVID-19 Symptoms During the Third Wave in Non-vaccinated Subjects and Effects of Hesperidin Therapy: A randomized, double-blinded, placebo-controlled study. medRxiv.

[16] United States Department of Agriculture: Food and Nutrition Service Child and Adult Care Food Program: Ensuring Children and Adults Have Access to Nutritious Meals and Snacks. https://www.fns.usda.gov/cacfp.

[17] United States Department of Agriculture U.S. Summary, FY 2020–FY 2021—Generated from National Data Bank Version 8.2 PUBLIC on 11/12/2021 August 2021. https://fns-prod.azureedge.net/sites/default/files/data-files/keydata-august-2021.pdf.

[18] Stephens L., Rains C., Benjamin-Neelon S.E. (2021). Connecting Families to Food Resources amid the COVID-19 Pandemic: A Cross-Sectional Survey of Early Care and Education Providers in Two U.S. States. Nutrients.

[19] Filip R., Anchidin-Norocel L., Gheorghita R., Savage W.K., Dimian M. (2021). Changes in Dietary Patterns and Clinical Health Outcomes in Different Countries during the SARS-CoV-2 Pandemic. Nutrients.

[20] Silverman J.R., Wang B.Z. (2021). Impact of School Closures, Precipitated by COVID-19, on Weight and Weight-Related Risk Factors among Schoolteachers: A Cross-Sectional Study. Nutrients.

[21] Bemanian M., Mæland S., Blomhoff R., Rabben Å.K., Arnesen E.K., Skogen J.C., Fadnes L.T. (2021). Emotional Eating in Relation to Worries and Psychological Distress Amid the COVID-19 Pandemic: A Population-Based Survey on Adults in Norway. Int. J. Environ. Res. Public Health.

[22] Barker M.E., Thompson K.A., McClean S.I. (1996). Do Type As Eat Differently? A Comparison of Men and Women. Appetite.

[23] Zhang J., Zhang Y., Huo S., Ma Y., Ke Y., Wang P., Zhao A. (2020). Emotional Eating in Pregnant Women during the COVID-19 Pandemic and Its Association with Dietary Intake and Gestational Weight Gain. Nutrients.

